# Investigational medications in 9,638 hospitalized patients with severe COVID-19: lessons from the “fail-and-learn” strategy during the first two waves of the pandemic in 2020

**DOI:** 10.1186/s13037-023-00358-9

**Published:** 2023-04-11

**Authors:** Adam C. Delgado, Brendon Cornett, Ye Ji Choi, Christina Colosimo, Vincent P. Stahel, Oliwier Dziadkowiec, Philip F. Stahel

**Affiliations:** 1 Department of Surgery, Sky Ridge Medical Center, Lone Tree, CO 80124 USA; 2 Graduate Medical Education, HCA Healthcare Continental Division, Denver, CO 80237 USA; 3 University of Colorado, Boulder, CO 80309 USA; 4 Mission Health, HCA Healthcare North Carolina Division, Asheville, NC 28803 USA; 5 Department of Surgery, Brody School of Medicine, East Carolina University, Greenville, NC 27858 USA; 6 Department of Specialty Medicine, College of Osteopathic Medicine, Rocky Vista University, Parker, CO 80134 USA

**Keywords:** COVID-19, Corticosteroids, Remdesivir, Azithromycin, Hydroxychloroquine, Tocilizumab, Mechanical ventilation, Mortality.

## Abstract

**Background:**

The early surge of the novel coronavirus disease 2019 (COVID-19) pandemic introduced a significant clinical challenge due to the high case-fatality rate in absence of evidence-based recommendations. The empirical treatment modalities were relegated to historical expertise from the traditional management of acute respiratory distress syndrome (ARDS) in conjunction with off-label pharmaceutical agents endorsed under the “emergency use authorization” (EUA) paradigm by regulatory agencies. This study was designed to evaluate the insights from the “fail-and-learn” strategy in 2020 before the availability of COVID-19 vaccines and access to reliable insights from high-quality randomized controlled trials.

**Methods:**

A retrospective, multicenter, propensity-matched, case-control study was performed on a data registry comprising 186 hospitals from a national health care system in the United States, designed to investigate the efficacy of empirical treatment modalities during the early surge of the COVID-19 pandemic in 2020. Reflective of the time-windows of the initial two surges of the pandemic in 2020, patients were stratified into “Early 2020” (March 1–June 30) versus “Late 2020” (July 1–December 31) study cohorts. Logistic regression was applied to determine the efficacy of prevalent medications (remdesivir, azithromycin, hydroxychloroquine, corticosteroids, tocilizumab) and supplemental oxygen delivery modalities (invasive vs. non-invasive ventilation) on patient outcomes. The primary outcome measure was in-hospital mortality. Group comparisons were adjusted for covariates related to age, gender, ethnicity, body weight, comorbidities, and treatment modalities pertinent to organ failure replacement.

**Results:**

From a total of 87,788 patients in the multicenter data registry screened in this study, 9,638 patients were included who received 19,763 COVID-19 medications during the first two waves of the 2020 pandemic. The results showed a minimal, yet statistically significant, association with hydroxychloroquine in “Early 2020” and remdesivir in “Late 2020” with reduced odds of mortality (odds ratios 0.72 and 0.76, respectively; *P* = 0.01). Azithromycin was the only medication associated with decreased odds of mortality during both study time-windows (odds ratios 0.79 and 0.68, respectively; *P* < 0.01). In contrast, the necessity for oxygen supply showed significantly increased odds of mortality beyond the effect of all investigated medications. Of all the covariates associated with increased mortality, invasive mechanical ventilation had the highest odds ratios of 8.34 in the first surge and 9.46 in in the second surge of the pandemic (*P* < 0.01).

**Conclusion:**

This retrospective multicenter observational cohort study on 9,638 hospitalized patients with severe COVID-19 revealed that the necessity for invasive ventilation had the highest odds of mortality, beyond the variable effects observed by administration of the prevalent EUA-approved investigational drugs during the first two surges of the early 2020 pandemic in the United States.

## Background

When the novel coronavirus disease 2019 (COVID-19) was declared a global pandemic on March 11, 2020, there was limited scientific understanding of the pathophysiology of viral transmission, mechanisms of infection, and pathology of disease [1–4]. In the absence of high-level scientific evidence, early medical treatment strategies for COVID-19 were relegated to empirical off-label pharmaceutical agents and anecdotal expertise from the traditional management of acute respiratory distress syndrome (ARDS) caused by other infectious agents [5–8]. The U.S. Food and Drug Administration (FDA) granted the emergency use authorization (EUA) to a variety of investigational drugs that were outside of the scope of the original approved indication for the treatment of patients with severe COVID-19 [9–15], including pharmacological agents designed for the treatment of rheumatic and autoimmune diseases [16–18]. These include remdesivir, an antiviral drug for the treatment of Ebola virus disease [19]; tocilizumab, a monoclonal antibody blocking the human interleukin-6 receptor which was originally developed as an immunosuppressant in patients with rheumatoid arthritis [20–22]; the broad-spectrum antibiotic azithromycin which also exerts antiviral activity [23]; and the anti-malaria drug hydroxychloroquine [24–26]. As the pandemic progressed through 2020, it became increasingly evident that a younger cohort of patients suffered from adverse outcomes due to persistent hyperinflammation and thromboembolic complications which were attributed to severe immune system dysregulation in response to the viral infection [27–30]. This notion led to the resurgence of corticosteroids for COVID-19 patients at risk of respiratory deterioration during the second wave of the pandemic [31–35]. However, the efficacy of these investigational compounds remained largely equivocal due to the empirical nature of their application in absence of robust evidence from well-designed clinical trials during the early phase of the pandemic in 2020.

Now at three years into the novel coronavirus pandemic, this study was designed to determine the lessons learned from the use of prevalent EUA-approved medications during the early waves of the novel coronavirus pandemic in 2020, based on a large-scale retrospective analysis from a national healthcare system in the Unites States.

## Methods

A retrospective, multicenter, propensity-matched, case-control study was designed based on a data registry comprising 186 hospitals from a national health care system in the United States (HCA Healthcare, Nashville, TN). The denominator of the study population consisted of all adult inpatients of 18–89 years of age with a COVID-19 diagnosis verified by a positive SARS-CoV-2 polymerase chain reaction (PCR) test admitted to an intensive care unit (ICU) from March 1, 2020, to December 31, 2020. Exclusion criteria consisted of protected populations, pregnant women, minors < 18 years of age, elderly patients ≥ 90 years of age, and patients with documented autoimmune diseases. Patients who did not receive any of the specific COVID-19 medications investigated in this study (remdesivir, azithromycin, hydroxychloroquine, corticosteroids, tocilizumab) were excluded from analysis. The included patient population was stratified into the first wave (“Early 2020” patient cohort; March 1–June 30, 2020) and the second wave (“Late 2020” patient cohort; July 1–December 31, 2020). The unequal time-windows of 4 months (“Early 2020”) vs. 6 months (“Late 2020”) were pragmatically selected to reflect on the approximate timing of the first two main surges of the COVID-19 pandemic in the United States. The primary outcome measure was in-hospital mortality. Patients stratified into the “Early 2020” versus “Late 2020” cohorts were propensity matched at a ratio of 1:1 using the following covariates: race, gender, body mass index (BMI), diabetes mellitus, hypertension, liver disease, congestive heart failure, mechanical ventilation, continuous renal replacement therapy (CRRT), and extracorporeal membrane oxygenation (ECMO). “Invasive ventilation” was defined by endotracheal intubation or tracheostomy, whereas all other modalities of adjunctive oxygen supply were defined as “non-invasive ventilation” (e.g. nasal cannula, BiPAP, CPAP, etc.). Logistic regression was used to model mortality as the primary outcome. Separate logistic regression models were used for each of the following COVID-19 medications: remdesivir, azithromycin, hydroxychloroquine, corticosteroids, and tocilizumab. The odds ratios and confidence intervals for each medication were compared between the time periods for overlap. No significant overlap between the time periods were detected to indicate effect modification. The *greedy nearest neighbor matching* methodology was used with a caliper width of 0.2 standard deviations. In addition, double adjustment propensity scoring was used to allow matching variables in the main analysis. A *P*-value of 0.05 was considered statistically significant. This study was reviewed by the HCA Healthcare Institutional Review Board (IRB) and was deemed exempt from IRB oversight (ID# 2021 − 117).

## Results

**Fig. 1:**
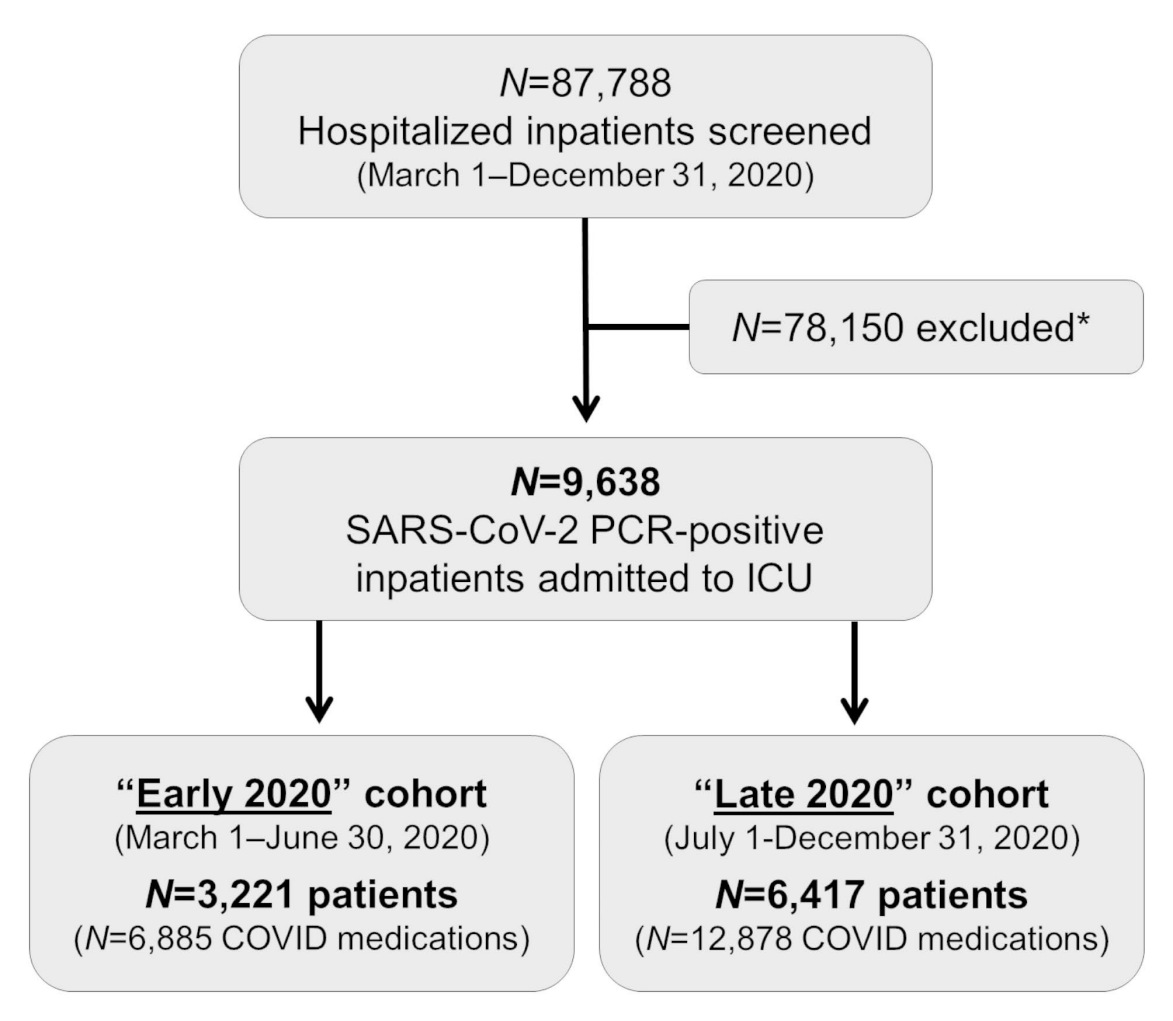
Patient selection flowchart Legend: *Exclusion criteria listed in the methods section Abbreviations: COVID, novel coronavirus disease; SARS-CoV-2, severe acute respiratory syndrome coronavirus 2; PCR, polymerase chain reaction

A total of 87,788 patients in the multicenter data registry were screened for inclusion criteria during the study time-window from March 1–December 31, 2020. Of these, 78,150 patients were excluded from analysis based on the following exclusion criteria: Negative SARS-CoV-2 PCR test (n = 10,845); no ICU admission (n = 54,311); no COVID-19 medication (n = 2,583); no propensity matching score (n = 5,271); protected populations (n = 2,159); missing data elements (n = 2,820); autoimmune disease (n = 85); pregnancy (n = 76). The patient selection flowchart is shown in Fig. 1. After exclusion criteria, the final population included in this study consisted of 9,638 patients who received 19,763 COVID-19 medications during the first two waves of the pandemic. Of these, 3,221 patients (“Early 2020”) and 6,417 patients (“Late 2020”) were treated with 6,885 and 12,878 COVID-19 medications, respectively, averaging two COVID-19 medications per patient during both study periods. The patients’ demographic data, stratified by medication type, are shown in Table 1. The specific metrics related to payor source, admission and discharge disposition are shown in Table 2. 44% of patients died during their ICU stay, 41% were discharged to home, and 13% were discharged to an inpatient rehabilitation facility (Table 2). The majority of patients received corticosteroids (88%), followed by azithromycin (53%), remdesivir (42%), hydroxychloroquine (13%), and tocilizumab (8%). The medication treatment modalities and odds ratios between the “Early 2020” vs. “Late 2020” study cohorts are shown in Tables 3 and 4, respectively.

**Table 1:** Patient demographic data, stratified by medication type

	Azithromycin	Cortico-steroids	Hydroxy-chloroquine	Remdesivir	Tocilizumab	Total
Number of patients	5,102	8,498	1,239	4,124	800	9,638
Gender						
Female	1,984 (38.9%)	3,324 (39.1%)	472 (38.1%)	1,546 (37.5%)	259 (32.4%)	3,781 (39.2%)
Male	3,118 (61.1%)	5,174 (60.9%)	767 (61.9%)	2,578 (62.5%)	541 (67.6%)	5,857 (60.8%)
**Ethnicity**						
Asian	226 (4.4%)	298 (3.5%)	66 (5.3%)	159 (3.9%)	34 (4.3%)	350 (3.6%)
Black	770 (15.1%)	1,241 (14.6%)	220 (17.8%)	524 (12.7%)	105 (13.1%)	1,455 (15.1%)
Hispanic	9 (0.2%)	15 (0.2%)	2 (0.2%)	7 (0.2%)	3 (0.4%)	16 (0.17%)
Other	1,295 (25.4%)	2,023 (23.8%)	277 (22.4%)	1,063 (25.8%)	262 (32.8%)	2,298 (23.8%)
Natives	21 (0.4%)	32 (0.4%)	5 (0.4%)	13 (0.3%)	3 (0.4%)	35 (0.4%)
White	2,781 (54.5%)	4,889 (57.5%)	669 (54.0%)	2,358 (57.2%)	393 (49.1%)	5,484 (56.9%)
**Age**						
Median (IQR)	66 (55–75)	67 (56–76)	67 (56–75)	65 (55–74)	63 (54–73)	67 (56–76)
Range	18–89	18–89	24–89	18–89	18–89	18–89
**BMI**						
Median (IQR)	29 (26–34)	29 (26–34)	29 (26–34)	30 (26–35)	30 (26–34)	29 (25–34)
Range	15–45	15–45	15–45	15–45	17–45	15–45

**Table 2:** Admission and discharge metrics

	Azithromycin (N = 5,102)	Cortico-steroids (N = 8,498)	Hydroxy-chloroquine (N = 1,239)	Remdesivir (N = 4,124)	Tocilizumab (N = 800)	Total (N = 9,638)
Admission Source						
Direct Admit from Home	4,516 (88.51%)	7,233 (85.11%)	1,063 (85.79%)	3,587 (86.98%)	714 (89.25%)	8,209 (85.17%)
Interfacility Transfer	586 (11.49%)	1,265 (14.89%)	176 (14.21%)	537 (13.02%)	86 (10.75%)	1,429 (14.83%)
**Admission Quarter (2020)**						
Q1	365 (7.15%)	180 (2.12%)	375 (30.27%)	14 (0.34%)	19 (2.38%)	451 (4.68%)
Q2	1,698 (33.28%)	2,102 (24.74%)	782 (63.12%)	820 (19.88%)	530 (66.25%)	2,770 (28.74%)
Q3	1,555 (30.48%)	3,035 (35.71%)	60 (4.84%)	1,442 (34.97%)	232 (29.00%)	3,142 (32.60%)
Q4	1,484 (29.09%)	3,181 (37.43%)	22 (1.78%)	1,848 (44.81%)	19 (2.38%)	3,275 (33.98%)
**Discharge Disposition**						
Expired	1,967 (38.55%)	3,824 (45.00%)	543 (43.83%)	1,665 (40.37%)	433 (54.13%)	4,193 (43.50%)
Home	2,347 (46.00%)	3,329 (39.17%)	487 (39.31%)	1,834 (44.47%)	271 (33.88%)	3,907 (40.54%)
Transfer Out	170 (3.33%)	229 (2.69%)	46 (3.71%)	125 (3.03%)	30 (3.75%)	279 (2.89%)
Inpatient Rehab	618 (12.11%)	1,116 (13.13%)	163 (13.16%)	500 (12.12%)	66 (8.25%)	1,259 (13.06%)

**Table 3:** **Medication treatment modalities by study cohort.** The medication numbers do not match the respective patient cohort sizes due to an overlap of multiple medications applied in individual patients

Number of patients per cohort	“Early 2020” (*n* = 3,221)	“Late 2020” (*n* = 6,417)	Total (*N* = 9,638)
Number of patients per medication			
**Azithromycin**	2,063 (64.05%)	3,039 (47.36%)	5,102 (52.94%)
**Corticosteroids**	2,282 (70.85%)	6,216 (96.87%)	8,498 (88.17%)
**Hydroxychloroquine**	1,157 (35.92%)	82 (1.28%)	1,239 (12.86%)
**Remdesivir**	834 (25.89%)	3,290 (51.27%)	4,124 (42.79%)
**Tocilizumab**	549 (17.04%)	251 (3.91%)	800 (8.30%)

**Table 4:** **Logistic regression analysis of medication odds ratios for “Early 2020”** vs. **“Late 2020” study cohorts.** A *P*-value < 0.05 was considered statistically significant

	Odds Ratio	95% Confidence Interval		*P*-Value
Azithromycin “Early 2020”	0.79	0.65	0.95	0.01
Azithromycin “Late 2020”	0.68	0.56	0.82	< 0.01
Corticosteroids “Early 2020”	1.28	1.03	1.59	0.02
Corticosteroids “Late 2020”	1.36	0.74	2.48	0.32
Hydroxychloroquine “Early 2020”	0.72	0.59	0.88	< 0.01
Hydroxychloroquine “Late 2020”	1.10	0.49	2.48	0.82
Remdesivir “Early 2020”	1.21	0.98	1.51	0.08
Remdesivir “Late 2020”	0.76	0.62	0.93	0.01
Tocilizumab “Early 2020”	1.49	1.17	1.90	< 0.01
Tocilizumab “Late 2020”	1.01	0.62	1.64	0.98

**Fig. 2:**
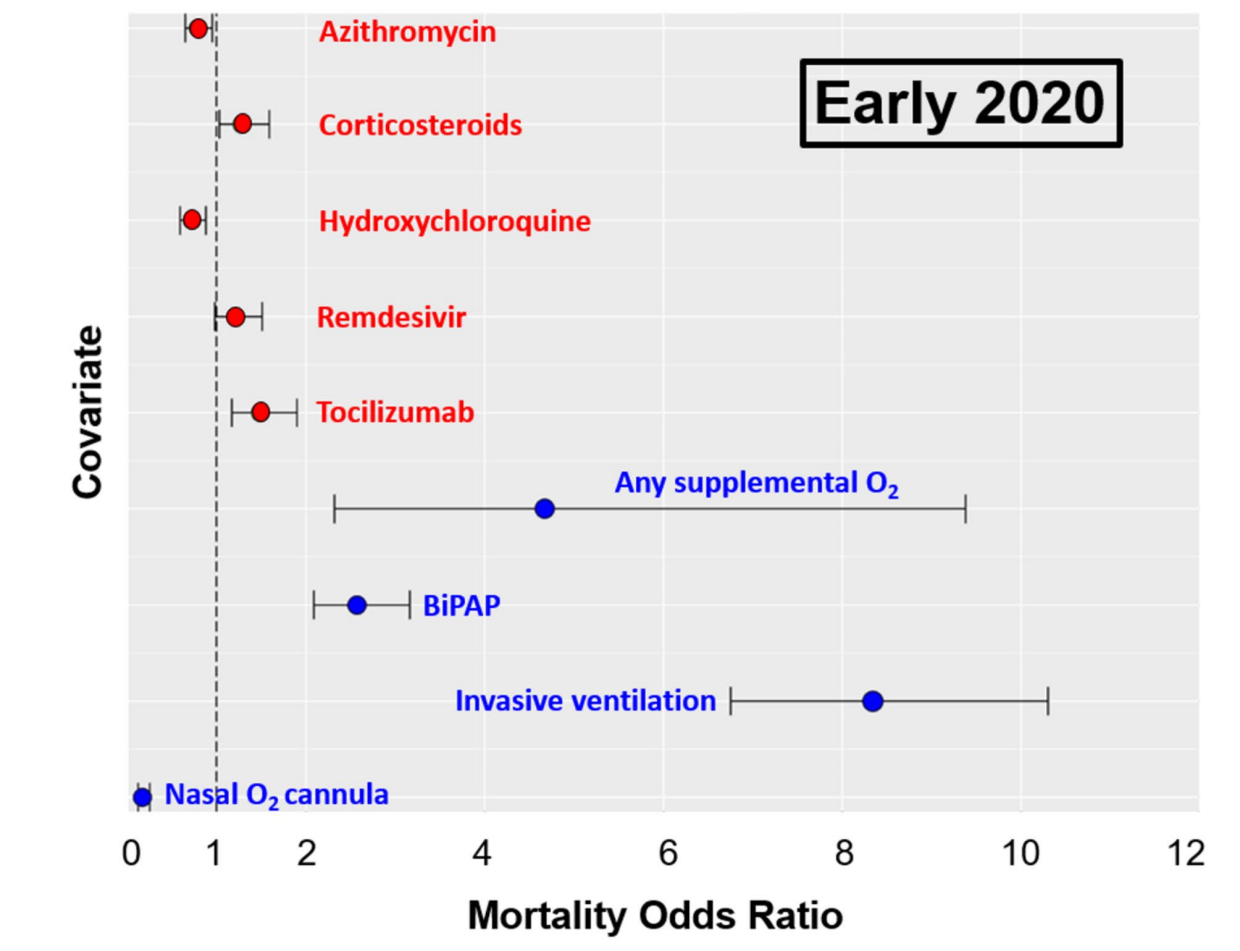
Forrest Plot of mortality odds ratios in the “Early 2020” cohort, stratified by medication and supplemental oxygen delivery modality Odds ratios below 1 indicate decreased odds of mortality Odds ratios above 1 indicated increased odds of mortality

**Fig. 3:**
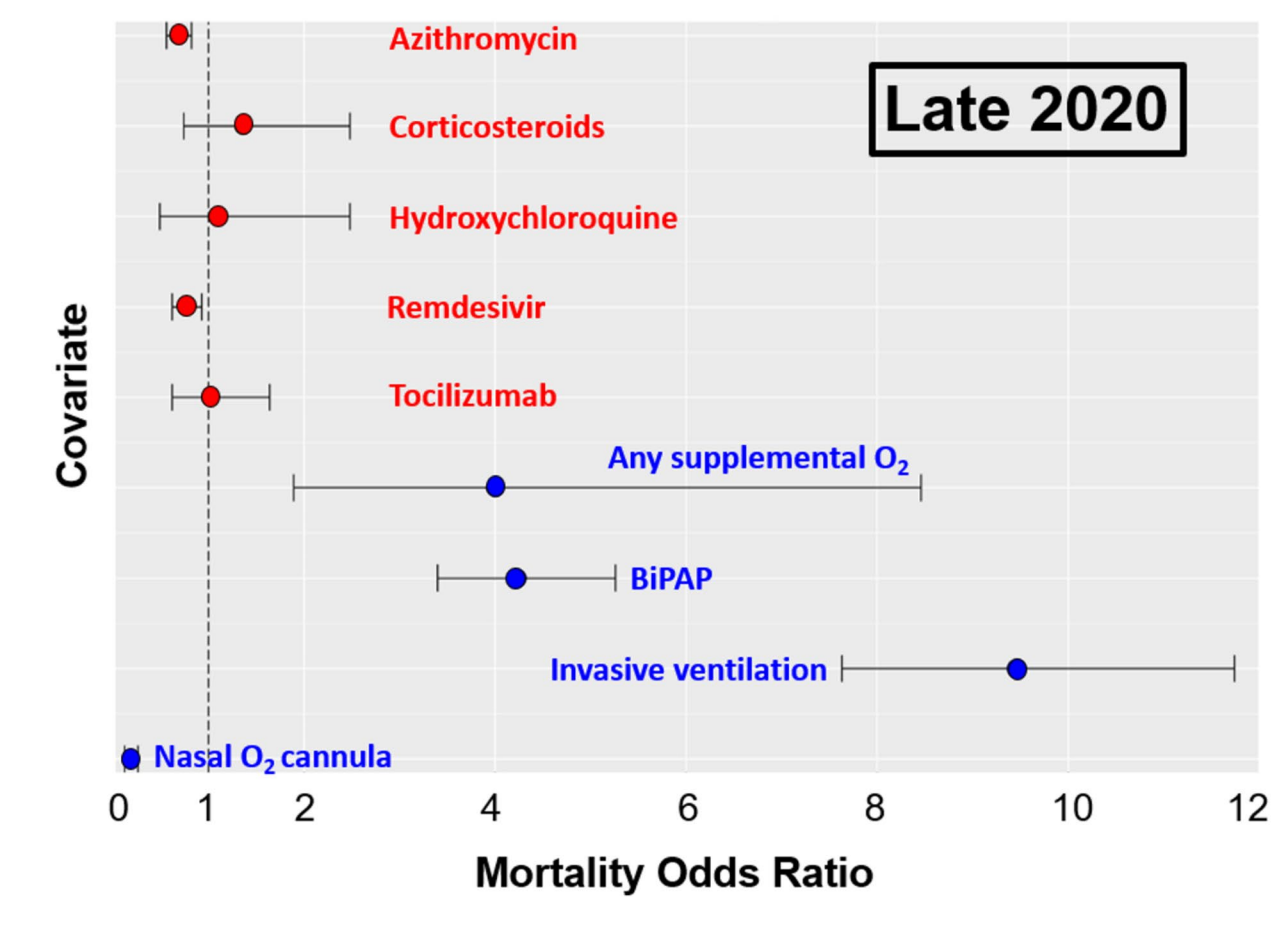
Forrest Plot of mortality odds ratios in the “Late 2020” cohort, stratified by medication and supplemental oxygen delivery modality Odds ratios below 1 indicate decreased odds of mortality Odds ratios above 1 indicated increased odds of mortality

As shown in Figs. 2 and 3, the odds of mortality from COVID-19 were significantly decreased in patients treated with azithromycin, both during the “Early 2020” (odds ratio: 0.79; CI: 0.65–0.95, *P* = 0.01) and “Late 2020” (odds ratio: 0.68; CI: 0.56–0.82, *P* < 0.01) time periods. Similarly, the administration of hydroxychloroquine in “Early 2020” (odds ratio: 0.72; CI: 0.59–0.88, *P* < 0.01) and remdesivir in “Late 2020” (odds ratio: 0.76; CI: 0.62–0.93, *P* = 0.01) were associated with significantly lower odds of mortality. The odds ratio of mortality in patients treated with hydroxychloroquine or remdesivir were significantly different between the two time periods of the pandemic in 2020 (Figs. 2 and 3). In contrast, the administration of corticosteroids during both time periods revealed significantly increased odds of mortality (odds ratios of 1.28 and 1.36; CI 1.03–1.59 and 0.74–2.48, respectively; *P* = 0.02). Similarly, patients treated with tocilizumab had a significantly increased likelihood of dying during the “Early 2020” pandemic surge (odds ratio: 1.49; CI: 1.17–1.90, *P* < 0.01), whereas tocilizumab appeared to have almost no effect on mortality in the “Late 2020” time period (odds ratio: 1.01; CI: 0.62–1.64, n.s.).

Since multiple confounding variables may affect mortality during the first two surges of the COVID-19 pandemic, we performed a logistic regression analysis for selected covariates related to age, body weight, ethnicity, comorbidities, and treatment modalities pertinent to the management of pulmonary and renal organ function. The covariates and respective odds ratios are shown in Table 5 (“Early 2020”) and Table 6 (“Late 2020”). Of all the significant covariates associated with increased mortality, invasive mechanical ventilation had the highest odds ratios of 8.34 in “Early 2020” (CI: 6.75–10.3, *P* < 0.01) and 9.46 in “Late 2020” (CI: 7.63–11.74, *P* < 0.01). The specific oxygen delivery modalities, stratified by medications given, are depicted in Table 7.

**Table 5:** **“Early 2020” logistic regression covariates pertinent to patient demographics, comorbidities, and selected pulmonary and renal organ management modalities.** A *P*-value < 0.05 was considered statistically significant. Abbreviations: BiPAP, bilevel positive airway pressure; BMI, body mass index; CRRT, continuous renal replacement therapy; ECMO, extracorporeal membrane oxygenation; O_2_, oxygen

	Odds Ratio	95% Confidence Interval		*P*-Value
Age	1.07	1.06	1.08	< 0.01
Elixhauser Comorbidity Index	1.12	1.07	1.17	< 0.01
Asian vs. white ethnicity	0.41	0.24	0.68	< 0.01
Black vs. white ethnicity	0.54	0.41	0.71	< 0.01
Hispanic vs. white ethnicity	0.66	0.07	6.72	0.76
Multiracial vs. white ethnicity	0.64	0.51	0.81	< 0.01
Native American vs. white ethnicity	1.18	0.25	5.67	0.68
BMI	0.99	0.97	1.00	0.13
Coagulopathy	1.49	1.18	1.87	< 0.01
Dementia	1.48	1.10	2.00	< 0.01
Psychosis	0.74	0.48	1.15	0.14
Cancer	1.67	1.09	2.56	0.04
Surgical procedure	0.39	0.27	0.58	< 0.01
Any O_2_ administration	4.67	2.32	9.38	< 0.01
Nasal O_2_ cannula	0.17	0.12	0.25	< 0.01
BiPAP	2.57	2.09	3.16	< 0.01
Invasive ventilation	8.34	6.75	10.3	< 0.01
CRRT	3.65	2.64	5.06	< 0.01
ECMO	3.16	1.21	8.24	0.02

**Table 6:** **“Late 2020” logistic regression covariates pertinent to patient demographics, comorbidities, and selected pulmonary and renal organ management modalities.** A *P*-value < 0.05 was considered statistically significant. Abbreviations: BiPAP, bilevel positive airway pressure; BMI, body mass index; CRRT, continuous renal replacement therapy; ECMO, extracorporeal membrane oxygenation; O_2_, oxygen

	Odds Ratio	95% Confidence Interval		*P*-Value
Age	1.06	1.05	1.07	< 0.01
Elixhauser Comorbidity Index	1.16	1.11	1.22	< 0.01
Asian vs. white ethnicity	0.56	0.32	0.97	0.04
Black vs. white ethnicity	0.61	0.45	0.81	< 0.01
Hispanic vs. white ethnicity	1.63	0.16	16.49	0.63
Multiracial vs. white ethnicity	0.83	0.65	1.05	0.16
Native American vs. white ethnicity	0.32	0.05	1.91	0.23
BMI	0.97	0.95	0.99	< 0.01
Coagulopathy	1.66	1.31	2.12	< 0.01
Dementia	1.88	1.38	2.55	< 0.01
Psychosis	0.80	0.49	1.31	0.37
Cancer	2.04	1.32	3.15	< 0.01
Surgical procedure	0.48	0.33	0.68	< 0.01
Any O_2_ administration	4.00	1.89	8.46	< 0.01
Nasal O_2_ cannula	0.18	0.12	0.26	< 0.01
BiPAP	4.23	3.40	5.26	< 0.01
Invasive ventilation	9.46	7.63	11.74	< 0.01
CRRT	2.53	1.80	3.55	< 0.01
ECMO	1.63	0.74	3.61	0.17

**Table 7:** **Supplemental oxygen delivery modalities, stratified by medications.** Abbreviations: BiPAP, bilevel positive airway pressure; CPAP, continuous positive airway pressure; ECMO, extracorporeal membrane oxygenation

	Azithro-mycin		Cortico-steroids		Hydroxy-chloroquine		Remdesivir		Tocilizumab		
	Early (N = 2539)	Late (N = 5341)	Early (N = 2711)	Late (N = 11,050)	Early (N = 1436)	Late (N = 143)	Early (N = 1013)	Late (N = 5852)	Early (N = 650)	Late (N = 451)	Total (N = 9,638)
**Non-Invasive Ventilation Measures**	2,303 (90.71%)	4,917 (92.06%)	2,516 (92.81%)	10,232 (92.60%)	1,299 (90.46%)	137 (95.80%)	972 (95.95%)	5,601 (95.71%)	614 (94.46%)	433 (96.01%)	8,709 (90.36%)
Nasal Oxygen Cannula	2,200 (86.65%)	4,709 (88.17%)	2,416 (89.12%)	9,805 (88.73%)	1,241 (86.42%)	135 (94.41%)	945 (93.29%)	5,401 (92.29%)	586 (90.15%)	401 (88.91%)	8,375 (86.90%)
Oxygen Mask	1,290 (50.81%)	2,635 (49.34%)	1,602 (59.09%)	5,917 (53.55%)	809 (56.34%)	72 (50.35%)	651 (64.26%)	3,370 (57.59%)	446 (68.62%)	305 (67.63%)	4,935 (51.20%)
BiPAP	583 (22.96%)	2,485 (46.53%)	880 (32.46%)	5,245 (47.47%)	244 (16.99%)	72 (50.35%)	372 (36.72%)	3,070 (52.46%)	249 (38.31%)	283 (62.75%)	2,955 (30.66%)
CPAP	171 (6.73%)	622 (11.65%)	272 (10.03%)	1,491 (13.49%)	86 (5.99%)	18 (12.59%)	119 (11.75%)	833 (14.23%)	71 (10.92%)	65 (14.41%)	872 (9.05%)
**Invasive Ventilation**	1,233 (48.56%)	2,509 (46.98%)	1,515 (55.88%)	5,699 (51.57%)	871 (60.65%)	89 (62.24%)	536 (52.91%)	3,026 (51.71%)	444 (68.31%)	316 (70.07%)	4,833 (50.15%)
Tracheo-stomy	66 (2.60%)	176 (3.30%)	97 (3.58%)	394 (3.57%)	40 (2.79%)	10 (6.99%)	44 (4.34%)	212 (3.62%)	22 (3.38%)	21 (4.66%)	303 (3.14%)
**ECMO**	14 (0.55%)	28 (0.52%)	33 (1.22%)	101 (0.91%)	15 (1.04%)	0 (0.00%)	23 (2.27%)	48 (0.82%)	10 (1.54%)	7 (1.55%)	106 (1.10%)

**Declarations**

## Discussion

This large-scale multicenter study on 9,638 hospitalized patients with severe COVID-19 was designed to evaluate lessons learned from empirical treatment modalities with five prevalent EUA-approved investigational drugs during the first two surges of the pandemic in 2020. The results show somewhat inconclusive data pertinent to the impact of these medications on patient outcomes during the “early” (March 1-June 30) and “late” (July 1 – December 31) surges of the 2020 pandemic. In contrast to the variable effect of these medications, the necessity for oxygen supply was associated with significantly increased odds of mortality beyond the effect of the five selected EUA-approved medications (Fig. 2). The requirement for any delivery modality for supplemental oxygen, reflective of patients who develop respiratory distress, was associated with a significantly increased odds of mortality by a factor of 4.67 in the “Early 2020” cohort, and 4.0 in the “Late 2020” cohort. Non-invasive ventilation using BiPAP was associated with a decreased risk of mortality in patients who required supplemental oxygen. In contrast, invasive mechanical ventilation was associated with the highest odds of mortality in both study time-periods, with dramatically increased odds of 8.34 in the “Early 2020” cohort, and 9.46 in the “Late 2020” cohort. These findings reflect on the early experience in the acute management of COVID-19 patients with acute respiratory failure which demonstrated that the early endotracheal intubation with mechanical ventilation was associated with increased mortality, leading to more judicial indications of invasive versus non-invasive ventilation strategies due to the detrimental lessons learned during the early phase of the pandemic, when patients in respiratory distress were more liberally intubated for mechanical ventilation [36, 37].

The five representative EUA-approved medications investigated in this study had less of an impact on mortality compared to the necessity for and modality of oxygen delivery (Fig. 2). Nevertheless, it was interesting to observe differences in mortality odds ratios between the individual medications and the two different time-windows of the pandemic surges in 2020. Remdesivir treatment did not show a significant association to mortality in surge 1, however, was linked to a significant decrease in mortality in surge 2. This may be due to the more widespread access to the antiviral drug, as suggested by the increase in the number of remdesivir administration from 834 in “Early 2020” to 3,290 in “Late 2020”. Azithromycin has seen a widespread anecdotal application during the early phase of the pandemic, however, its indication for the treatment of hospitalized patients with COVID-19 remains controversial and scientifically unsubstantiated [23]. In the current study, about half of our patients received azithromycin which was associated with a minimal, yet statistically significant, decrease in the odds of mortality during both time-windows in 2020. Our data are in contrast to a published systematic review and meta-analysis of the published literature in 2021 which included 16 trials in a total of 22,984 patients [23]. The study demonstrated no difference in mortality between COVID-19 patients treated with or without azithromycin [overall odd ratio: 0.95 (0.79–11.13)]. The authors concluded that the results from their meta-analysis do not support the use of azithromycin in the management of COVID-19 [23].

The anti-malaria drug hydroxychloroquine has received early global attention as a potential medication in COVID-19 due to promising preliminary insights from small scale studies [26]. However, the anecdotal “hype” around this medication was never supported by strong clinical evidence from better quality trials [26]. In our study, the number of patients treated with hydroxychloroquine decreased dramatically between the first and second surges, from 36% down to only 1.28%. This observation likely reflects the lack of efficacy and risk of significant side effects by the drug which became more widely understood after the first wave of the pandemic [24, 38]. In support of this notion, our study showed no relevant benefit in mortality between the two surges, with a large confidence interval for hydroxychloroquine in surge 2, likely attributed to the low sample size in the “Late 2020” cohort. Our data showed an overall increase in corticosteroid use. During the first surge, about 71% of patients admitted to the ICU were given corticosteroids, compared to 97% of hospitalized COVID-19 patients during the 2nd surge. The widespread administration of corticosteroids to nearly 100% of all patients makes trends on mortality difficult to interpret, as both survivors and non-survivors would have been treated with steroids. The controlled open-label RECOVERY trial investigated different modalities and dose regimens for dexamethasone administration in hospitalized COVID-19 patients, and demonstrated a decreased mortality in patients receiving invasive ventilation at 28 days [39]. In this landmark trial on 6,425 patients, corticosteroids were thought to reduce inflammatory damage to lungs and reduce progression to respiratory failure and death [39]. Subsequent to the publication of the RECOVERY trial, there has been widespread global use of corticosteroids for hospitalized COVID-19 patients with signs of respiratory distress. Our study was unable to determine a significant and sustained protective effect of corticosteroids during the two pandemic surges, likely attributed to the shortcoming discussed above. Similar to steroids, tocilizumab has strong anti-inflammatory properties as a neutralizing monoclonal antibody against the human IL-6 receptor. Early studies during the first surge of the pandemic showed a benefit of tocilizumab by reducing mortality in critically ill COVID-19 patients suffering from hyperinflammation and respiratory failure [40, 41]. However, these beneficial effects postulated for tocilizumab were refuted in subsequent large-scale studies which failed to demonstrate the efficacy of tocilizumab in reducing mortality in critically ill patients with COVID-19 [42, 43]. In our study, patients that required invasive mechanical ventilation had a higher likelihood of being treated with tocilizumab than those who did not require invasive ventilation. This phenomenon is likely due to the widespread notion during the first surge that tocilizumab was beneficial for critically ill COVID-19 patients with systemic hyperinflammation in terms of reducing ventilator-dependent days and improving patient outcomes. Later publications demonstrated that the lack of efficacy by tocilizumab is likely due to an underrecognized anti-inflammatory effect of IL-6 [44]. Our current understanding of the immune pathophysiology suggests that the blockade of the IL-6 receptor will lead to a redundant massive increase of other pro-inflammatory cytokines which will subsequently exacerbate the “cytokine release syndrome” (CRS) and worsen the extent of harmful systemic hyperinflammation [44]. Our current data demonstrate a decrease in the number of patients receiving tocilizumab between the first and second surge. This observation is likely reflective of the change in evidence and clinical recommendations in the peer-reviewed literature around the time of the second surge of the pandemic [45, 46].

Overall, we interpret the variability in beneficial effects of the five prevalent medications investigated in this study during the first and second surge by the notion that each individual medication likely had a lesser impact on the pathophysiology of COVID-19 than other confounding variables. Most importantly, the requirement for supplemental oxygen in patients with respiratory distress and the early intubation and mechanical ventilation in patients with respiratory failure appears to represent the highest independent predictor of mortality during both pandemic surges in 2020 (Fig. 2). This insight had changed the management of oxygen delivery strategies dramatically due to the lessons learned from the first surge of the pandemic, from early proactive airway management by endotracheal intubation and mechanical ventilation, towards leveraging safer non-invasive ventilatory strategies [36, 37, 47].

Another confounding factor in our current study is represented by the observation that most patients received at least two COVID-19 medications simultaneously, which decreases the ability to determine the impact of a single medication with a higher level of statistical certainty. The large sample size of 9,638 patients ensured the equal distribution of underlying demographic variables and risk factors for severe COVID-19 and adverse outcomes, including higher age, male gender, obesity, and ethnicity (Table 1). The patient population included in this study also showed a similar distribution of risk factors related to preexisting comorbidities, as determined by the Elixhauser Comorbidity Index (Tables 5 and 6). Finally, the selected patient cohort managed by temporary organ replacement support for salvage therapy by CRRT (for acute renal failure) or ECMO (for refractory respiratory failure in spite of maximized mechanical ventilation efforts) showed significantly higher odds of mortality during both surges of the pandemic in 2020 (Tables 5 and 6). Unequivocally, the observation that severely ill patients “in extremis” who require last-effort rescue strategies by CRRT or ECMO will have a higher predicted mortality than the cohort of COVID-19 patients without acute organ failure, represents a predictable platitude. Nevertheless, it is worth emphasizing that both sub-cohorts of CRRT and ECMO patients had significantly decreased odds of dying compared to patients with invasive mechanical ventilation in absence of these rescue modalities, with odds ratios of 3.65 and 2.53 for CRRT; 3.16 and 1.63 for ECMO; versus 8.34 and 9.46 for invasive ventilation, during the “Early 2020” and “Late 2020” study time windows, respectively (Tables 5 and 6). For example, the protocolized adherence to the guidelines by the “Extracorporeal Life Support Organization” (ELSO) for stringent patient selection for ECMO cannulation was associated with significantly improved outcomes from venous-venous ECMO salvage therapy in critically ill COVID-19 patients [48, 49]. These findings imply a judicious and standardized patient selection process for COVID-19 rescue therapies across the 186 hospitals and thereby emphasize the benefits of protocolized care “at scale” across a large healthcare system in the United States.

There are several methodological shortcomings and limitations to this study. First, the definition applied to the “early” and “late” time-windows of the pandemic is arbitrary and prone to potential misinterpretation of the data. While the unequal time-windows of 4 months (“Early 2020”) vs. 6 months (“Late 2020”) had been intentionally selected to reflect on the approximate timing of the first two main COVID-19 surges in the United States, this pragmatic selection does not account for differences in geographical “tiers” as the pandemic evolved in the United States. For example, participating hospitals in Florida will have seen different patient volumes and acuity during the “early” time-window of this study compared to participating hospitals in California, Texas, or Colorado. Conversely, southeastern states, such as Florida, were more dramatically affected from surging COVID-19 patient volumes during the second wave of the 2020 pandemic. Finally, the unequal arbitrary stratification of the time-windows into 4 vs. 6 months does not allow for a direct comparison of respective patient volumes and treatment numbers. Furthermore, our data source from the 87,788 patients included in the multicenter data registry did not allow to retrieve specific information pertaining to convalescent SARS-CoV-2 plasma due to the incorporation of this treatment modality within the overall fresh frozen plasma transfusion numbers in the blood bank data. Thus, we were unable to attribute any potential survival benefit in this patient cohort to the transfusion of convalescent plasma. Last but not least, this study suffers from the classic flaws of retrospective analysis of “large data” registries. In general, large population-based databases provide high statistical power to determine effect estimates over smaller single center registries, however, at the shortcoming of being unable to retrieve granular level data that would allow to investigate a direct causative relation between treatment and outcomes. Although the methodology applied propensity matching to make the medication groups more comparable, we cannot rule out that differences in mortality between medication groups were due to other confounding variables, since patients were not randomly assigned to medication treatment groups. Nevertheless, this retrospective multicenter cohort study allows to provide a high-level “lesson learned” on the efficacy of investigational off-label medications and on the impact of oxygen delivery modalities on the mortality and adverse outcome during the early wave of the COVID-19 pandemic in the United States.

## Conclusion

This retrospective multicenter observational cohort study on 9,638 hospitalized patients with severe COVID-19 revealed that the necessity for invasive ventilation had the highest odds of mortality, beyond the variable effects observed by administration of the prevalent EUA-approved investigational drugs during the first two surges of the early 2020 pandemic in the United States.

## Data Availability

Please contact the authors for specific data requests.
